# Seoul Virus and Hantavirus Disease, Shenyang, People’s Republic of China

**DOI:** 10.3201/eid1502.080291

**Published:** 2009-02

**Authors:** Yong-Zhen Zhang, Xue Dong, Xin Li, Chao Ma, Hai-Ping Xiong, Guang-Jie Yan, Na Gao, Dong-Mei Jiang, Ming-Hui Li, Lu-Ping Li, Yang Zou, Alexander Plyusnin

**Affiliations:** Chinese Center for Disease Control and Prevention, Beijing, People’s Republic of China (Y.-Z. Zhang, C. Ma, H.-P. Xiong, N. Gao, Y. Zou); Shenyang Center for Disease Control and Prevention, Shenyang, People’s Republic of China (X. Dong, X. Li, M.-H. Li); Shenhe District Center for Disease Control and Prevention, Shenyang (G.-J. Yan, D.-M. Jiang); Shenyang Infectious Disease Hospital, Shenyang (L.-P. Li); Haartman Institute, University of Helsinki, Helsinki, Finland (A. Plyusnin)

**Keywords:** HFRS outbreak, hantavirus, Seoul virus, laboratory rats, wild Norway rats, China, research

## Abstract

An outbreak of hemorrhagic fever with renal syndrome (HFRS) occurred among students in Shenyang Pharmaceutical University in 2006. We conducted a study to characterize etiologic agents of the outbreaks and clarify the origin of hantaviruses causing infections in humans and laboratory animals. Immunoglobulin (Ig) M or IgG antibodies against Seoul virus (SEOV) were detected in the serum samples of all 8 patients. IgG antibodies against hantavirus were also identified in laboratory rats, which were used by these students for their scientific research. Phylogenetic analysis showed that partial small segment sequences recovered from humans, laboratory rats, and local wild rats belonged to SEOV. Hantavirus sequences recovered from humans and laboratory rats clustered within 1 of 3 lineages of SEOV circulating among local wild rats in Shenyang. These results suggest that the HFRS outbreak in Shenyang was caused by SEOV that was circulating among local wild rats and had also infected the laboratory rats.

## Abstract 2

This outbreak was caused by the virus circulating in local wild rats through infection of laboratory rats.

Hantaviruses, members of the family *Bunyaviridae*, genus *Hantavirus*, cause 2 human zoonoses, hemorrhagic fever with renal syndrome (HFRS) in Asia and Europe and hantavirus pulmonary syndrome in North and South America (*1*). In their natural hosts, rodents of the families Muridae *and* Cricetidae, hantaviruses cause chronic infection with no apparent harm (2,3). HFRS has been recognized as a serious public health problem in China since 1955 (*4**,**5*). The disease is caused mainly by the *Hantaan virus* (HTNV), transmitted by the striped field mouse (*Apodemus agrarius*), and Seoul virus (SEOV), transmitted by the brown Norway rat (*Rattus norvegicus*) (*4**,**6*).

Transmission of hantaviruses among rodents and from rodents to humans generally occurs through inhalation of aerosolized excreta (*7*). HFRS outbreaks have occurred among farmers and workers during close contact with infected rodents in disease-endemic areas. Hantavirus infections have also occurred among technicians and researchers after handling laboratory rodents. The first report showed that contact with hantavirus-infected laboratory rats caused a HFRS outbreak among 13 doctors and 1 veterinarian at medical research institutions in Japan (*8*). Since 1975 and 1978, laboratory animal-associated HFRS outbreaks have been reported in several countries (*9**–**14*). Dozens of hantavirus infections in laboratory animals also occurred during the 1980s in China (*15*). Furthermore, 16 HFRS cases associated with laboratory rats occurred in 1983 in the Shanxi province (*16*). However, only a few reports have attempted to characterize the etiologic agents of the outbreaks and clarify the origin of hantaviruses causing infections in humans and laboratory animals (*9**,**17*).

Shenyang City (the capital of Liaoning Province) is located in northeastern China. Shenyang has always been one of the most seriously affected areas in China since the first outbreak of HFRS in 1958 (*5**,**18*). A total of 470 HFRS cases were reported in Shenyang in 2005; most of these cases occurred among farmers in the suburbs and the rural areas of Shenyang. Previous studies have shown the presence of 2 hantaviruses carried by rodents: HTNV, carried by striped field mice, and SEOV, carried by Norway rats in Shenyang (*18*). Serologic and genetic analyses suggest that the HFRS outbreak was caused by transmission of SEOV, which was circulating among local wild rats; the wild rats passed the virus to laboratory rats, which then infected humans. Our study characterizes etiologic agents of these outbreaks among students and clarifies the origin of hantaviruses causing infections in humans and laboratory animals.

## Materials and Methods

### Patients and Serum Samples

HFRS cases were defined by a national standard of clinical criteria and confirmed by detecting antibodies against hantavirus in serum samples obtained in 2006. Serum samples were collected from patients with clinical signs of HFRS and sent to the Shenyang Center for Disease Control and Prevention (Shenyang CDC) for detection of hantavirus-reactive antibodies, and then to the Institute for Communicable Disease Control and Prevention, Chinese Center for Disease Control and Prevention for further serologic and genetic characterization. Shenyang CDC conducted the HFRS epidemiologic studies. Information such as the date of onset of illness, fever, living conditions, history of exposure in dormitory and field, and clinical symptoms and signs was obtained and recorded.

### Laboratory Rats and Mice

All laboratory rats (Wistar) and mice (BALB/c) housed in the same animal facility in a pharmaceutical laboratory building were obtained from the Laboratory Animal Center of Shenyang Pharmaceutical University and were sampled. These rodents were generally >6 months of age and had been in the animal facility for >1 month. Serum and lung tissue samples were collected from all laboratory animals, placed in vials, stored immediately at –196^o^C, and transported to the laboratory for processing.

### Trapping of Rodents

During 2006–2007, wild rodents were captured on the grounds of the animal facility in the pharmaceutical laboratory building, in the vicinity of the laboratory Animal Center of Shenyang Pharmaceutical University during 1 month after the outbreak in 2006, and in a major HFRS-endemic focus in the suburbs of Shenyang in the autumn of 2006 and the spring of 2007 using snap-traps baited with peanuts. Lung tissue samples were taken from dissected animals, placed immediately into vials and stored at –196^o^C and, then transported to a laboratory for processing.

### Serologic Assays

Human serum samples were tested for immunoglobulin (Ig) G and IgM antibodies against HTNV and SEOV by indirect immunofluorescent assay (IFA). Serum samples from laboratory rodents were tested for IgG antibodies to SEOV or HTNV. IgG and IgM IFAs were performed with HTNV (strain 76–118)– and SEOV (strain L99)–infected Vero E6 cells. Cells were spread onto slides, air-dried, and fixed with acetone. Samples were serially diluted in 2-fold steps in phosphate-buffered saline, starting with the initial dilution of 1:2, then added to the cells, and incubated for 90 min at 37°C. Slides were washed in phosphate-buffered saline and incubated with fluorescein isothiocyanate (FITC)–labeled rabbit antihuman IgG and IgM antibodies (Sigma, St. Louis, MO, USA), which are gamma-chain– and mu-chain specific, respectively, at 37°C for 30 min. For rodent samples, FITC-labeled goat antimouse or antirat IgG was used. IgG titers >40 and IgM titers >20 were considered positive.

### Detection of Hantavirus Antigen

**Figure 1:**
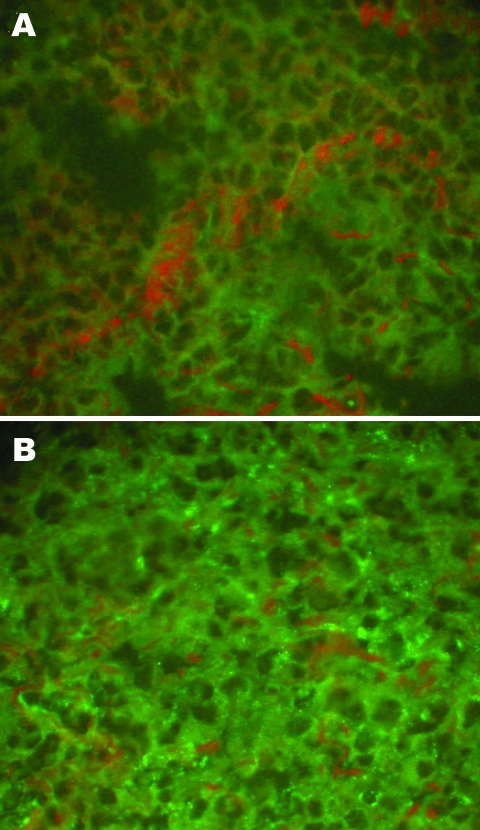
Detection of hantaviral antigens by indirect immunofluorescent assay. A) Hantaviral antigen- negative *Rattus norvegicus* lung tissue, detected with anti-L99 and 76-118 hantavirus sera. B) Hantaviral antigen-positive *R. norvegicus* lung tissue, detected with anti-L99 and 76-118 hantavirus antibodies. Magnification ×400.

Viral antigens in the lung tissue (frozen sections) of rats and mice were detected by using indirect IFA as described previously (*19*), with rabbit anti-SEOV/L99 and HTNV/76–118 hantavirus antibodies and FITC-labeled goat antirabbit IgG (Sigma). Scattered, granular fluorescence in the cytoplasm was considered a positive reaction (Figure 1).

### Reverse Transcription–PCR (RT–PCR) and Sequencing

Total RNA was extracted from rodent lung tissues by using the TRIzol reagent (Invitrogen, Carlsbad, CA, USA), according to the manufacturer’s instructions and subjected to RT-PCR for amplification of partial hantavirus small (S) segment sequences. cDNA was synthesized with avian myeloblastosis virus reverse transcriptase (Promega, Beijing, China) in the presence of primer P14 (*20*). Partial S-segment sequences of SEOV (nt 620–999) were amplified from SEOV by using primers HV-SFO and HV-SRO for initial PCR (*21*), and primers SEO-SF and SEOV-SR for the second round of amplification (*22*). For amplification of partial S-segment sequence (nt 514–1,026) from HTNV, the same primer pair HV-SFO/HV-SRO was used for initial PCR and the primer pair HSF /HSR was used for nested PCR (*22*).

The PCR products (380 bp and 513 bp, respectively) were gel-purified by using QIAquick Gel Extraction kit (QIAGEN, Beijing, China) according to the manufacturer’s instructions and cloned into the pMD18-T vector (TaKaRa, Dalian, China). The ligated products were transformed into JM109-competent cells. DNA sequencing was performed with the ABI-PRISM Dye Termination Sequencing kit and an ABI 373-A genetic analyzer (Applied Biosystems, Carlsbad, CA, USA). At least 2 cDNA clones were used to determine each viral sequence. In case of discrepancy, a third cDNA clone was sequenced.

### Phylogenetic Analysis

**Figure 2:**
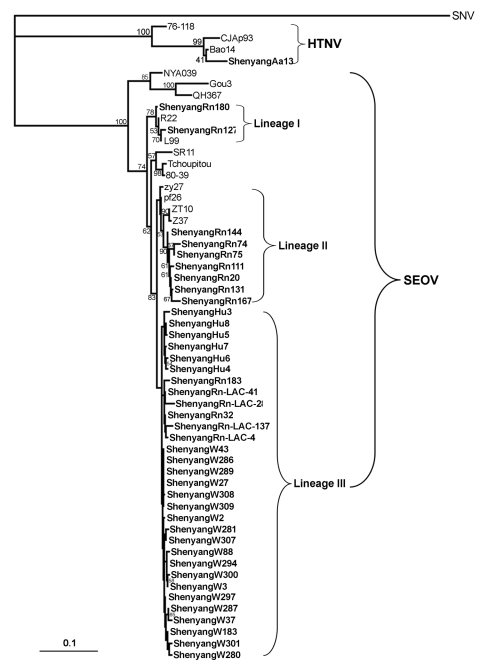
Phylogenetic tree of hantaviruses based on partial sequences of the small (S) segment (nt 600–999 for Seoul virus (SEOV) and nt 514–1026 for hantaan virus (HTNV). PHYLIP program package (3.65) was used to construct the phylogenetic trees by using the neighbor-joining (NJ) method and the maximum likehood (ML) with 1,000 replicates. The tree, constructed by using the ML method, had a similar topology as that constructed by the NJ method (data not shown). Bootstrap values were calculated from 1,000 replicates; only values >50% are shown at the branch nodes. The sequence of Sin Nombre virus (SNV) was used as an outgroup. Partial S-segment sequences recovered from 6 patient serum samples were designated ShenyangHu3, ShenyangHu4, ShenyangHu5, ShenyangHu6, ShenyangHu7, and ShenyangHu8. Sequences from *Rattus norvegicus* trapped in 2006 in the vicinity of the Laboratory Animal Center of Shenyang Pharmaceutical University were designated ShenyangRn-LAC-4, ShenyangRn-LAC-28, and ShenyangRn-LAC-41. ShenyangRn-LAC-137, from *R. norvegicus* and *A.* *agrarius,* trapped in 2006–2007 in the major hemorrhagic fever with renal syndrome–endemic focus in the rural areas of Shenyang were designated ShenyangRn20, ShenyangRn32, ShenyangRn74, ShenyangRn75, ShenyangRn111, ShenyangRn127, ShenyangRn131, ShenyangRn144, ShenyangRn167, ShenyangRn180, ShenyangRn183, and ShenyangAa13), from hantavirus antigen–positive laboratory rats were designated ShenyangW–. Sequences obtained in this study are shown in **boldface**. The GenBank accession numbers of the other partial S segment sequences are SNV/NM H10 (L25748); HTNV/76–118 (M14626), HTNV/CJAp93 (EF208953), HTNV/Bao14 (AB127998); SEOV/NYA039 (EF210131), SEOV/Gou3 (AF288651), SEOV/QH367 (DQ081717), SEOV/SR11 (M34881), SEOV/Tchoupitoulas (AF329389), SEOV/80–39 (AY273791), SEOV/L99 (AF488708), SEOV/R22 (AF488707), SEOV/pf26 (AY006465), SEOV/zy27 (AF406965), SEOV/Z37 (F187082), and SEOV/ZT10 (AY766368). Scale bar represents genetic distance.

The PHYLIP program package version 3.65 (http://evolution.genetics.washington.edu/phylip.html) was used to construct phylogenetic trees by using the neighbor-joining method with 1, 000 bootstrap replicates. Alignments were prepared with ClustalW version 1.83 (www.ebi.ac.uk/Tools/clustalw2/index.html). The nucleotide identities were calculated by using the DNAStar program (DNASTAR, Madison, WI, USA). For comparison, hantavirus sequences were retrieved from GenBank (www.ncbi.nlm.nih.gov/Genbank) (Figure 2).

## Results

### Patients and Survey Results

From March 8 through April 22, 2006, symptoms of hantavirus infection developed in 8 postgraduate students (5 men and 3 women), who studied at Shenyang Pharmaceutical University located in the center of the Shenyang. All patients met the national clinical criteria of HFRS, required hospitalization, and were treated in Shenyang Infectious Hospital. Fever, proteinuria, and mild hemorrhagic complications were observed in all patients, but without the distinct clinical stages seen in the severe form of the disease caused by HTNV (Table 1). Other clinical symptoms such as weakness, backache, nausea, vomiting, abdominal pain, eyeball pain, and hypotension were not observed.

**Table 1:** Clinical symptoms and signs of HFRS patients in Shenyang, China, 2006*

Data	Patient no.							
1	2	3	4	5	6	7	8
Sex	F	F	F	M	M	M	M	M
Age, y	24	24	25	24	24	24	24	29
Signs and symptoms								
Fever	+	+	+	+	+	+	+	+
Weakness	+	+	–	+	+	–	+	+
Headache	+	+	–	+	–	+	+	+
Backache	+	–	+	+	–	+	+	+
Eyeball pain	+	–	–	–	+	–	+	–
Nausea	–	–	–	+	–	–	+	+
Vomiting	–	–	–	–	–	–	+	–
Abdominal pain	–	–	–	+	–	–	–	–
Hemorrhagic complications	+	+	+	+	+	+	+	+
Oligouria	+	+	–	+	–	–	+	–
Proteinuria	+	+	+	+	+	+	+	+
Hypotension	+	–	–	+	–	–	–	–

*HFRS, hemorrhagic fever with renal syndrome.

The 8 students lived in different rooms in the 2 dormitories on the university campus. They had no history of exposure to wild rats in their rooms. Notably, all their roommates had been in good health. Further, the students neither performed field studies nor had a history of exposure to rats or mice in the field during the previous 6 months. All 8 students conducted their research in the same department and had direct contact with a colony of laboratory rats and mice in the animal facility in the pharmaceutical laboratory building. Hantavirus infection did not develop in any person who did not have direct contact with the laboratory rats and mice.

### Serologic and Genetic Investigation of Patient Serum Samples

Serum samples from all 8 patients were collected at day 1 of hospitalization (2–4 days post onset of fever). Samples were tested for IgM and IgG antibodies by IFA using SEOV- or HTNV-infected cells (Table 2). All serum samples showed higher IgM and IgG titers in SEOV-specific IFA. In 6 of 8 serum samples, the IgG titers against SEOV were 4-fold higher; in the remaining 2 serum samples, the titers against SEOV were 2-fold higher (Table 2). These results suggested that the HFRS cases were caused by SEOV.

**Table 2:** Serologic analysis of samples from HFRS patients by indirect IFA, Shenyang, China, 2006*

Serum sample no.	IgM assay†			IgG assay†	
	SEOV	HTNV		SEOV	HTNV
1/06	40	20		640	160
2/06	40	20		320	80
3/06	40	–		320	80
4/06	20	–		320	160
5/06	40	–		320	80
6/06	40	20		160	20
7/06	40	20		320	80
8/06	40	–		320	160

*HFRS, hemorraghic fever with renal syndrome; IFA, immunofluorescent assay; Ig, immunoglobulin; SEOV, Seoul virus; HTNV, hantaan virus; –, could not be detected. †Numbers represent the endpoint titers of anti-hantavirus (SEOV or HTNV) antibodies in the patients’ serum samples.

Total RNA was extracted from all serum samples and analyzed by SEOV S-segment–specific or HTNV S segment–specific RT-PCR. Hantavirus genome sequences were amplified from 6 serum samples collected soon after the onset of disease by using SEOV S-segment–specific primers, not HTNV S segment–specific primers. That the HFRS cases were caused by SEOV was confirmed. Corresponding SEOV strains were designated ShenyangHu3, ShenyangHu4, ShenyangHu5, ShenyangHu6, ShenyangHu7, and ShenyangHu8.

### Analysis of Laboratory Rats and Mice

Serum samples from all suspected laboratory rats and mice were tested for IgG antibodies against SEOV or HTNV, and lung tissues were analyzed for the presence of hantavirus antigen by indirect IFA. Hantavirus antibodies were detected in 32 of 139 rats; the hantavirus antigen was detected in 26 of these 32 rats (designated ShenyangW–; Figure 2). Antibodies against HTNV or SEOV, or hantavirus antigen have not been observed in laboratory mice.

### Rodent Trapping and Analysis

To investigate whether SEOV strains identified in the patients and laboratory rats originated in the local wild rodent population, 156 Norway rats (*R. norvegicus*) were trapped in the major HFRS endemic focus during the autumn of 2006 and the spring of 2007 in the vicinity of the Laboratory Animal Center of Shenyang Pharmaceutical University. Four of 156 wild rats were found to be positive for hantavirus antigen by IFA. Hantavirus S-segment sequences were recovered from these animals (corresponding hantavirus strains were designated ShenyangRn-LAC-4, ShenyangRn-LAC-28, ShenyangRn-LAC-41, and ShenyangRn-LAC-137). No rodents had been caught in the pharmaceutical laboratory building, suggesting that the laboratory animal infection occurred in the Laboratory Animal Center.

A total of 299 rodents (56 striped field mice [*A.* *agrarius*] and 243 Norway rats [*R. norvegicus*]) were captured in 2006–2007 during the major HFRS endemic focus in the rural areas of Shenyang, which is ≈15 km from the Laboratory Animal Center. Of these rodents, 11 Norway rats and 1 striped field mouse were found to be positive for hantavirus antigen by IFA. Hantavirus S-segment sequences were recovered from these animals (corresponding hantavirus strains were designated ShenyangRn20, ShenyangRn32, ShenyangRn74, ShenyangRn75, ShenyangRn111, ShenyangRn127, ShenyangRn131, ShenyangRn144, ShenyangRn167, ShenyangRn180, ShenyangRn183, and ShenyangAa13).

### Genetic Analyses

Partial S-segment sequences were recovered from 6 patient serum samples, 19 laboratory rats (designated ShenyangW–, Figure 2); 15 wild Norway rats, and 1 striped field mouse trapped in the outbreak region. Genetic analysis showed that the partial S-segment sequences recovered from all humans, laboratory rats, and wild rats were very closely related to each other, with 95.6% to 99.8% sequence identity (Appendix Table). These sequences have a higher level of identity to SEOV (85.5–99.2%) than to HTNV and other hantavirus types. Further comparison showed that the partial S-segment sequences recovered from human and laboratory rats were very closely related to each other, with 98.7% to 99.8% sequence identity. The 5% nucleotide divergence among hantaviruses carried by wild rats suggested that perhaps>1 genetic lineage of SEOV co-circulated in Shenyang. Notably, the sequences of hantaviruses carried by humans and laboratory rats were more closely related to those recovered from the wild rats trapped in the vicinity of the Laboratory Animal Center (ShenyangRn-LAC-4, ShenyangRn-LAC-28, ShenyangRn-LAC-41, and Shenyang-LAC-137). Moreover, these sequences also shared a higher homology with those recovered from the lung tissue samples that were collected from the wild Norway rats trapped in the major HFRS endemic focus in the rural areas of Shenyang (ShenyangRn32 and ShenyangRn180).

As expected, the partial S-segment sequence recovered from 1 striped field mouse was closely related to those from HTNV. The sequence showed especially high identity (99.0%) to strain Bao14 isolated from *A.* *agrarius* in Heilongjiang (*23*), which is also in northeastern China.

### Phylogenetic Analyses

In the present study, phylogenetic analysis of partial S-segment sequences confirmed the molecular link between SEOV strains from patients, laboratory Norway rats, and the wild Norway rats trapped in the vicinity of the Laboratory Animal Center and the disease-endemic areas (Figure 2). As shown in Figure 2, all partial S-segment sequences from humans, laboratory rats, and wild rats fell into the SEOV genetic clade, well separated from other hantaviruses, thus indicating that the HFRS outbreak was caused by SEOV. Notably, the partial S sequences from wild rats were divided into 3 lineages. The partial S sequences recovered from humans and laboratory rats formed 2 groups, and the sequences derived from the wild rats trapped in the vicinity of the Laboratory Animal Center formed another group. Together, these 3 groups formed a lineage that also included the sequences ShenyangRn32 and ShenyangRn183, which were recovered from the wild rats trapped in the major HFRS-endemic focus in the rural areas of Shenyang. This suggests that the HFRS outbreak had been caused by strains belonging to this particular lineage of SEOV.

## Discussion

HFRS has been recognized as a serious problem in Shenyang since the first outbreak in 1958 (*18*). Despite comprehensive control measures, including vaccination, that have been carried out in the major endemic area of the city in the past several years, 361–630 HFRS cases have been reported annually from 2001 through 2005. Here we report the results of serologic and molecular epidemiologic investigation of a laboratory rats–associated outbreak of hantavirus disease involving 8 postgraduate students in Shenyang. The patients had clinical symptoms and biochemical findings typical of HFRS cases occurring in China. Serologic tests and the analysis of recovered hantavirus genome sequences showed that the outbreak was caused by a transmission of SEOV variants from the local wild Norway rats through the laboratory Norway rats to humans.

Serologic tests and phylogenetic analysis indicated that the HFRS cases were caused by the SEOV spread by laboratory rats. HFRS cases associated with laboratory-acquired infections have been reported in several countries (*8**–**13**,**16*). Notably, hantavirus infections were found to be more common in laboratory Norway rats than in mice and other laboratory animals (*8**,**9**,**12**–**15*). However, only a few investigations gave clear clues as to the origin of hantaviruses circulating in laboratory animals (*9**,**17*).

Previous studies have shown the presence of 2 hantaviruses carried by rodents: HTNV carried by the striped field mice and SEOV by the brown Norway rats in Shenyang (*18*). In the present study, serologic tests showed that all mouse serum samples were antihantavirus antibody-negative, and hantaviral antigens were not identified in the mouse lung tissues and HTNV-specific sequences were not detected in human serum samples. These results suggest that human infections were not caused by HTNV, although our data demonstrated that HTNV is circulating in *A.* *agrarius* in Shenyang. Both human and laboratory rat serum specimens were anti-SEOV antibody positive, which suggests that the infections were caused by SEOV. Due to the cross-reactivity of sera, exact serotyping for diagnoses of individual patients was not possible. Seroepidemiologic studies may sometimes misidentify the causative hantavirus if typing is based only on ELISA, IFA, or immunoblot analysis (*24*). Therefore, partial hantavirus S-segment sequences were amplified from the patient sera and laboratory rat lung tissues. All partial S-segment sequences recovered from 6 human and 26 laboratory rats belonged to SEOV; they were closely related to each other, and clustered together on the phylogenetic tree (Figure 2). These results confirmed that the HFRS outbreak in Shenyang was caused by SEOV and suggested the likely route of infection was from wild rats to laboratory rats and then to humans.

Analysis of wild rats trapped in the vicinity of human case-patients and the major HFRS epidemic focus allowed comparison of SEOV genome sequences in humans and rats (laboratory and wild). Phylogenetic analysis of the partial S-segment sequences indicated that 3 lineages of SEOV are co-circulating in wild rats in Shenyang (Figure 2). Notably, the sequences from patients and laboratory rats were clustered within 1 of these 3 lineages. Our results suggest that the viruses carried by the laboratory rats originated from the prevalent SEOV strains circulating in wild Norway rats in this area, and then were transmitted to humans.

In conclusion, our study indicates that the HFRS outbreak was caused by SEOV circulating in local wild Norway rats through laboratory rats. Because hantavirus infection in wild Norway rats is frequent in most regions of China (*5*), this study reinforces conclusion that vigilance is needed to prevent laboratory-associated cases of hantavirus disease.

## Supplementary Material

Appendix Table Partial small nucleotide and amino acid sequence identities of hantaviruses from Shenyang, China, with those of other hantaviruses % Identity Strain with strain*
