# Supplementary material for: Seoul Virus and Hantavirus Disease, Shenyang, People’s Republic of China
Source: Emerg Infect Dis. 2009 Feb;15(2):200–6. doi: 10.3201/eid1502.080291 (PMC2662651; doi:10.3201/eid1502.080291)
Supplement: Appendix Table — Partial small nucleotide and amino acid sequence identities of hantaviruses from Shenyang, China, with those of other hantaviruses % Identity Strain with strain* [file 08-0291_appT-s1.pdf]

Appendix Table. Partial small nucleotide and amino acid sequence identities of hantaviruses from Shenyang, China, with those of other hantaviruses

Strain	% Identity with strain*																				
	1	2	3	4	5	6	7	8	9	10	11	12	13	14	15	16	17	18	19	20	21
1. ShenyangRn127		99.8	95.0	95.3	95.0	95.0	95.8	95.5	95.5	96.1	96.1	95.8	96.1	99.2	95.0	96.1	87.1	68.9	66.8	66.3	69.2
2. ShenyangRn180	99.8		95.0	95.3	95.0	95.0	95.8	95.5	95.5	96.1	96.1	95.8	96.1	99.2	95.0	96.1	87.1	68.9	66.8	66.3	69.2
3. ShenyangRn131	99.2	99.2		99.7	96.8	96.8	97.6	97.1	97.4	97.4	97.4	97.1	97.4	95.0	94.5	97.9	85.5	67.9	66.6	65.3	68.9
4. ShenyangRn144	99.2	99.2	99.8		97.1	97.1	97.9	97.4	97.6	97.6	97.6	97.4	97.6	95.3	94.7	98.2	85.8	67.9	66.6	65.3	68.9
5. ShenyangHu3	99.8	99.8	99.2	99.2		99.5	99.2	98.7	98.9	98.9	98.9	99.2	98.9	94.7	95.0	98.4	86.1	67.1	65.3	64.2	68.9
6. ShenyangHu4	99.2	99.2	98.4	98.4	99.2		99.2	98.7	98.9	98.9	98.9	99.2	98.9	94.7	95.0	98.4	86.1	67.1	65.3	64.2	68.9
7. ShenyangRn32	99.8	99.8	99.2	99.2	99.8	99.2		99.5	99.7	99.7	99.7	99.5	99.7	95.5	95.8	99.2	86.3	67.6	65.8	64.7	68.9
8. ShenayngRn183	99.2	99.2	98.4	98.4	99.2	98.4	99.2		99.2	99.5	99.5	99.2	99.5	95.3	95.5	98.7	86.3	67.4	65.5	64.5	68.7
9. ShenyangRn-LAC-4	99.8	99.8	99.2	99.2	99.8	99.2	99.8	99.2		99.5	99.5	99.2	99.5	95.3	95.5	98.9	86.6	67.4	65.5	64.5	68.7
10. ShenyangRn-LAC-41	99.8	99.8	99.2	99.2	99.8	99.2	99.8	99.2	99.8		99.8	99.7	99.8	95.8	96.1	98.9	86.3	67.9	66.1	65.0	69.2
11. ShenyangW-43	99.8	99.8	99.2	99.2	99.8	99.2	99.8	99.2	99.8	99.8		99.7	99.8	95.8	96.1	98.9	86.3	67.9	66.1	65.0	69.2
12. ShenyangW-88	99.8	99.8	99.2	99.2	99.8	99.2	99.8	99.2	99.8	99.8	99.8		99.7	95.5	95.8	98.7	86.1	67.9	66.1	65.0	69.2
13. ShenyangW-280	99.8	99.8	99.2	99.2	99.8	99.2	99.8	99.2	99.8	99.8	99.8	99.8		95.8	96.1	98.9	86.3	67.9	66.1	65.0	69.2
14. L99	99.8	99.8	99.2	99.2	99.8	99.2	99.8	99.2	99.8	99.8	99.8	99.8	99.8		94.7	95.8	86.3	68.2	66.6	66.1	68.9
15. SR11	98.4	98.4	97.6	97.6	98.4	97.6	98.4	97.6	98.4	98.4	98.4	98.4	98.4	98.4		96.1	87.1	67.4	65.8	64.7	68.4
16. Zy27	99.2	99.2	98.4	98.4	99.2	98.4	99.2	98.4	99.2	99.2	99.2	99.2	99.2	99.2	97.6		86.3	67.4	65.5	64.5	68.7
17. Gou3	98.4	98.4	97.6	97.6	98.4	97.6	98.4	97.6	98.4	98.4	98.4	98.4	98.4	98.4	98.4	97.6		68.2	66.3	66.6	67.6
18. ShenyangAa13	69.0	69.0	69.0	69.0	69.0	68.3	69.0	68.3	69.0	69.0	69.0	69.0	69.0	69.0	68.3	68.3	68.3		95.3	94.7	87.1
19. Bao14	69.0	69.0	69.0	69.0	69.0	68.3	69.0	68.3	69.0	69.0	69.0	69.0	69.0	69.0	68.3	68.3	68.3	99.8		96.1	86.8
20. CJAp93	69.8	69.8	69.8	69.8	69.8	69.0	69.8	69.0	69.8	69.8	69.8	69.8	69.8	69.8	69.0	69.0	69.0	99.2	99.2		87.6
21. 76-118	69.8	69.8	69.8	69.8	69.8	69.0	69.8	69.0	69.8	69.8	69.8	69.8	69.8	69.8	69.0	69.0	69.0	98.4	98.4	97.6	

*Percent identities for nucleotide (above the diagonal) and amino acid (below the diagonal) sequences are presented.
